# Isolating *Escherichia coli* strains for recombinant protein production

**DOI:** 10.1007/s00018-016-2371-2

**Published:** 2016-10-11

**Authors:** Susan Schlegel, Pierre Genevaux, Jan-Willem de Gier

**Affiliations:** 1 Department of Environmental Systems Science, ETH Zürich, 8092 Zürich, Switzerland; 2 Laboratoire de Microbiologie et de Génétique Moléculaires, Centre de Biologie Intégrative (CBI), Université de Toulouse, CNRS, UPS, Toulouse, France; 3 Department of Biochemistry and Biophysics, Stockholm University, Svante Arrheniusväg 16C, 106 91 Stockholm, Sweden

**Keywords:** *Escherichia coli*, Recombinant protein, Protein production, Strain isolation, Evolution, Mutagenesis

## Abstract

*Escherichia coli* has been widely used for the production of recombinant proteins. To improve protein production yields in *E. coli*, directed engineering approaches have been commonly used. However, there are only few reported examples of the isolation of *E. coli* protein production strains using evolutionary approaches. Here, we first give an introduction to bacterial evolution and mutagenesis to set the stage for discussing how so far selection- and screening-based approaches have been used to isolate *E. coli* protein production strains. Finally, we discuss how evolutionary approaches may be used in the future to isolate *E. coli* strains with improved protein production characteristics.

## Introduction

By the end of the nineteenth century, the German microbiologist Theodor Escherich discovered a fast-growing bacterium that was called *Escherichia coli* after its discoverer. *E. coli* has become one of the most important model organisms in biology and the main workhorse in biotechnology. As a model organism, *E. coli* has been widely used to study evolution, and in biotechnology, it has been routinely used for the production of recombinant proteins [1]. However, recombinant protein production yields and the quality of the produced material are often not satisfactory. To create *E. coli* strains with improved protein production characteristics, directed engineering approaches, like deleting genes encoding proteases and co-expressing genes encoding molecular chaperones, have been commonly used [2]. However, thus far, there have been only few reported examples of the isolation of *E. coli* protein production strains using evolutionary, i.e., screening- and selection-based approaches. Aim of this review is to discuss the in-our-opinion most relevant examples. To set the stage for this, we first give an overview of some in-our-opinion critical basics of bacterial evolution and mutagenesis. Based on our own experience, such an overview is very useful if one is interested in evolving *E. coli* strains for protein production, but does not have a background in bacterial genetics. However, those who are familiar with bacterial evolution and mutagenesis may skip the overview and immediately go to the section ‘*E. coli* as a platform for the production of recombinant proteins’.

## Evolution of bacteria

Evolution is defined as the change in heritable traits of biological populations over successive generations and is a continuously ongoing process. At the basis of evolution are mutations, which are heritable changes in the DNA sequence that can be faithfully replicated. Thus, only a permanent change constitutes a mutation.

**Fig. 1:**
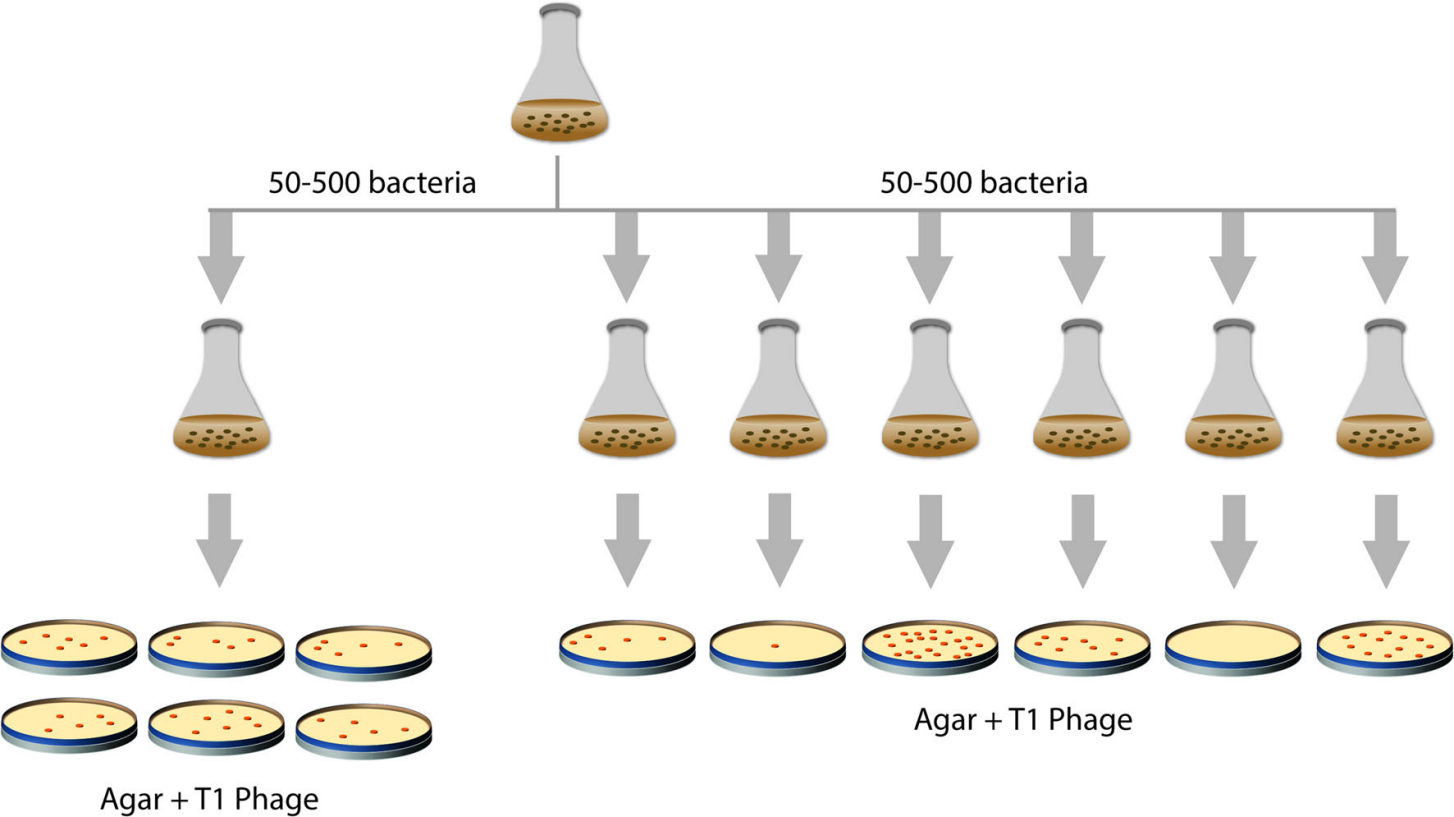
The Luria and Delbrück experiment. In 1943, Luria and Delbrück devised an experiment to address if mutations occur prior to selection or in response to it (‘mutation’ versus ‘acquired hereditary immunity’) [3]. Several aliquots from single *E. coli* cultures and from multiple, independent *E. coli* cultures were spread on plates containing bacteriophage T1 (‘virus α’). On these plates, only bacteria resistant (immune) to bacteriophage T1 survive and form colonies. This allowed estimating the number of bacteriophage T1 resistant bacteria in the cultures. In aliquots from the same culture, variation observed in the number of bacteriophage T1 resistant mutants was minor and could be attributed to experimental error. In contrast, the number of resistant mutants in aliquots of the multiple independent cultures varied greatly. Luria and Delbrück concluded that, in this setup, ‘resistance to virus is due to a heritable change of the bacterial cell which occurs independently of the action of the virus’ (cit. [3])

How do changes in heritable traits in bacteria occur? For a long time, it was not clear if bacteria somehow adapt to an environment by a process of directed change or if constantly spontaneous mutations occur that subsequently can be selected for. In 1943, Salvador Luria and Max Delbrück tested these two hypotheses, the random-mutagenesis hypothesis and the directed change hypothesis, in a landmark study [3] (Fig. 1). Luria and Delbrück used *E. coli* and the bacteriophage T1, which kills *E. coli*, as selective agent. However, mutations in the genes encoding the cell-envelope proteins TonA (a.k.a. FhuA) and TonB can make *E. coli* resistant against this bacteriophage [4]. In their study, Luria and Delbrück used (i) a single culture for spreading aliquots of *E. coli* cells on plates containing bacteriophage T1, and (ii) multiple independent cultures for spreading aliquots of *E. coli* cells on plates containing bacteriophage T1. Only bacteria resistant to bacteriophage T1 would survive and form colonies on the bacteriophage T1 containing plates, allowing estimating the number of bacteriophage T1 resistant bacteria in the aforementioned cultures. Using the single culture, the number of bacteriophage T1 resistant mutants in each aliquot was almost the same, whereas the number of resistant mutants in aliquots of the multiple independent cultures varied a lot. These results were in line with the random-mutagenesis hypothesis; i.e., mutations occur before selection rather than being induced by the selecting agent. In 1952, Esther and Joshua Lederberg showed that pre-existing mutations in bacteria that had never been exposed to an antibiotic could render them antibiotic-resistant [5], thus providing even more compelling evidence in support of the random-mutagenesis hypothesis.

## DNA integrity and mutagenesis

Being able to maintain the integrity of its DNA during replication and upon damage is key to *E. coli* survival. DNA replication is driven by DNA polymerases (P), and mistakes made by the DNAPs can introduce mutations. Also damage to DNA, i.e., a lesion, which can constitute a chemical alteration of a base, sugar or phosphate, can lead to mutations. In the following sections, we will give a succinct introduction to the different types of mutations and the major players involved in maintaining DNA integrity in *E. coli*, i.e., its DNAPs and DNA-repair systems. Finally, we will introduce the main global regulatory networks and some other important factors that can affect DNA integrity and mutagenesis in *E. coli*.

### Types of mutations

Mutations can, in theory, occur anywhere in the genome, and based on their effect on the fitness of the bacterium, they can either be neutral, deleterious or beneficial. It has been estimated that the majority of mutations (50–70 %) has no effect on fitness, 30–50 % are likely to be detrimental or lead to a complete loss of viability, and only very few mutations are expected to be beneficial (0.01–1 %) [6]. It should be kept in mind that a mutation that is beneficial under certain circumstances may be neutral or even have deleterious effects if conditions change.

**Fig. 2:**
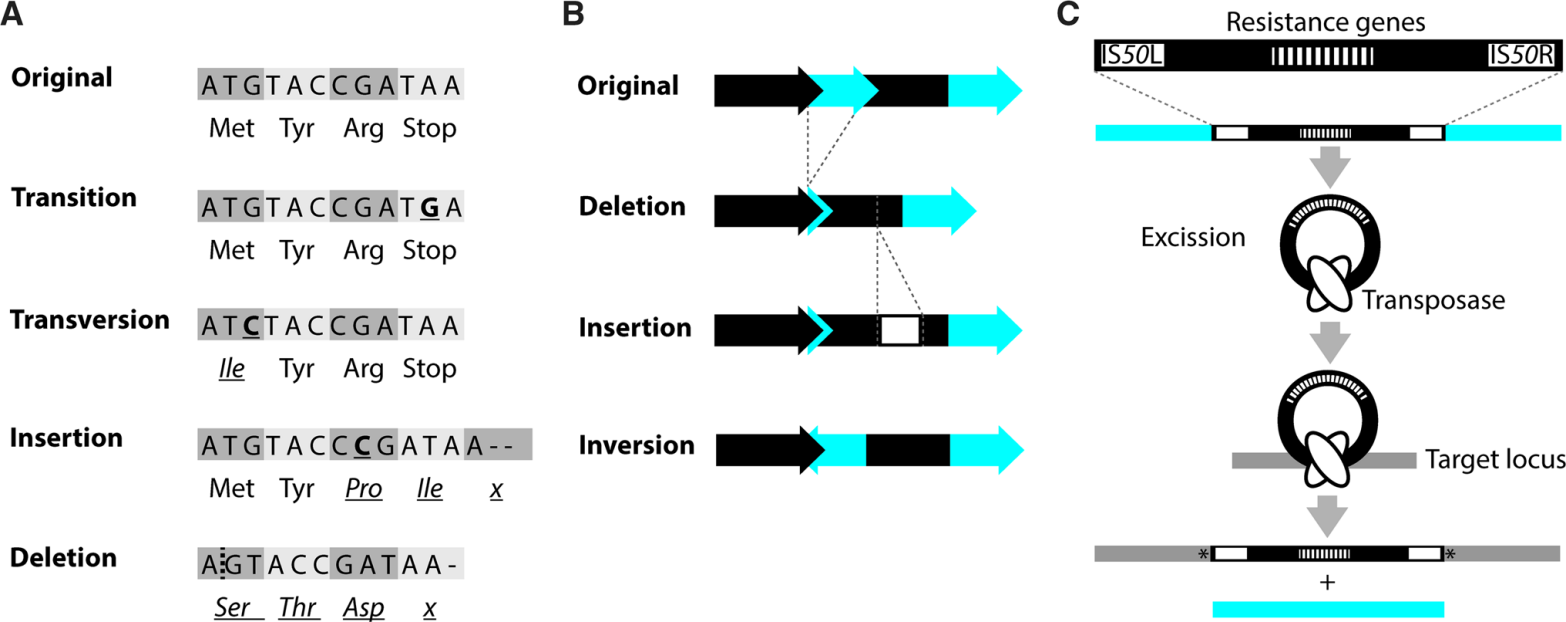
Types of mutations. Mutations can cause a large variety of changes in a genome. According to the nature of the change relative to the ancestral sequence, alterations may be grouped into base substitutions (i.e., transitions and transversions), insertions, deletions, inversions and translocations. **a** Examples of the possible effects of a single nucleotide alteration, including a nucleotide insertion and deletion, in a coding region. In this figure, the bases constitute codons and the encoded amino acids are indicated below the DNA sequence to illustrate possible effects. **b** Examples of larger-scale alterations. Genes are depicted as *arrows*, non-coding regions as *bars*. **c** Schematic representation of a transposition using the Tn*5* transposon as an example. Tn*5* is a composite transposon with two flanking IS*50* elements and contains multiple resistance genes [125]. A transposase (encoded by IS*50R*) mediates excision of Tn*5* from the donor locus and integration into a new location. In the target sequence, Tn*5* insertion leads to duplication of a few base pairs (indicated by *asterisk*). Note that transposition mechanisms differ depending on the transposable element The outline of figure 2c was taken from [156] with permission

At the sequence level, mutations are commonly grouped according to the nature of the change relative to the ancestral sequence into base substitutions, insertions, deletions, inversions and translocations [4] (Fig. 2a–c). In a base substitution, one nucleobase is exchanged for another. If a purine (adenine or guanine) is exchanged for the other purine or a pyrimidine (cytosine or thymine) is replaced by the other pyrimidine, the resulting change is called a base transition. In a transversion, the purines are changed into pyrimidines and the other way around. Base pair changes can occur as a result of internal factors like mis-pairing during replication, spontaneous deamination, or oxidation of bases by reactive oxygen species. External factors like irradiation or added chemicals can also induce base pair changes.

Deletions and insertions are often referred to as indels. In small indels, a single or several base pairs are removed or added to the DNA. Head-to-tail oriented repeats of the same base-pair units, so-called short tandem DNA repeats (STRs), are considered hotspots for the occurrence of small indels due to strand slipping and misalignment during DNA replication, or recombination events [7–9]. Hotspots are regions in the genome that are more likely to acquire mutations than others [10]. Larger deletions and insertions, as well as sizeable inversions that can affect entire genomic regions are primarily thought to be caused by recombination events between homologous regions, like rRNA genes, prophages, and transposable elements (i.e., transposons and IS-elements), that are present at several sites in the genome (e.g., [11–15]). However, as for STRs, even distant sequence repeats that are only a few nucleotides in length may lead to smaller and larger alterations (e.g., [8, 16, 17]). In this context, it is noteworthy that (some) transposable elements are flanked by short sequence repeats that may lead to multiplication or deletion of the transposable element itself [16, 18].

Rather than ‘passively’ promoting chromosomal alterations, the defining feature of transposable elements is their ability to translocate to another position in the genome (Fig. 2c). The mechanisms of transposition differ between the different elements, and the transposition event may lead to alterations in the DNA sequence of the donor locus, the target locus, or both [19, 20]. Notably, transposable elements seem to vary with respect to their target site specificity. Whilst some transposable elements seem to prefer certain DNA sequences, others, like Tn*5*, have not been connected to a specific integration site or sequence [20, 21].

### DNA polymerases

DNA replication is driven by DNA polymerases (DNAPs). Here, we give an overview of the five different DNAPs in *E. coli* and describe their roles in DNA replication and the occurrence of mutations. We refer to Fijalkowska et al. for a recent, comprehensive review on the different DNAPs in *E. coli* [22].

DNAP III is the main DNAP in *E. coli* and primarily responsible for synthesizing the leading and most of the lagging strand during DNA replication (e.g., [23]). The polymerase function is confined to the α-subunit of the enzyme, which is encoded by *dnaE* [24]. The DNAP III holoenyzme entails a proofreading function. If a wrong nucleotide is incorporated, it can immediately be removed by the 3′–5′ exonuclease activity of DNAP III. The exonuclease activity of DNAP III is confined to the ε subunit of the enzyme, which is encoded by *dnaQ/mutD* [25]. Mutations in e.g., *dnaQ/mutD* can considerably promote mutagenesis in *E. coli* and have been employed to facilitate the isolation of protein production strains [26, 27]. Also, DNAP I, which is encoded by *polA*, has a major role during DNA replication (e.g., [22]). DNAP I degrades RNA primers stemming from lagging-strand synthesis and re-fills the remaining gaps using the upstream Okazaki fragment as primer, and also participates in several repair pathways (see below). In contrast to DNAP III, DNAP I is a monomer that combines polymerase activity, and 3′–5′ as well as 5′–3′ exonuclease activities in the same polypeptide [28–30].

DNAP II is encoded by *dinA* and combines polymerase activity and 3′–5′ exonuclease activity in one protein [31, 32]. It has been suggested that DNAP II participates in a variety of processes related to DNA integrity, including DNA replication under lenient conditions and the general response to DNA damage, the so-called SOS response (e.g., [22, 33]). DNAP II is also able to bypass small lesions in the DNA, thereby maintaining DNA replication at the risk of promoting mutations (e.g., [34]). This ability of DNAP II and others has been termed translesion DNA synthesis [35] and can be essential to keep DNA replication going when repair pathways either fail to recognize lesions or if there are too many lesions to be processed [33] (see below).

The primary role of DNAP IV (encoded by *dinB*) and DNAP V (encoded by *umuC*/*D*), is to ensure DNA replication under stressful conditions [33]. However, deletion of *dinB* has also been shown to decrease the number of small frameshift mutations and base substitutions under standard conditions [36]. Expression of the genes encoding DNAP IV and V is induced as part of the SOS response [37]. Both DNAPs are able to bypass certain DNA lesions, and due to a lack of proofreading activity, DNA synthesis by both enzymes is essentially error-prone, but to different extents [38, 39].

### Repairing single-strand DNA lesions

In *E. coli*, the base excision repair (BER), methyl-directed mismatch repair (MMR), very short patch repair (VSR), nucleotide excision repair (NER), and transcription-coupled repair (TCR) pathways are all involved in repairing damage affecting one of the two DNA strands. Mutations in several of the involved factors (see below) have been associated with mutator phenotypes, thus illustrating their importance for maintaining DNA integrity [40]. In this section, we will give a brief overview of the most basic features of these DNA repair pathways. To do justice to the impressive body of work in this area, we will point the reader to some excellent reviews for further information.

**Fig. 3:**
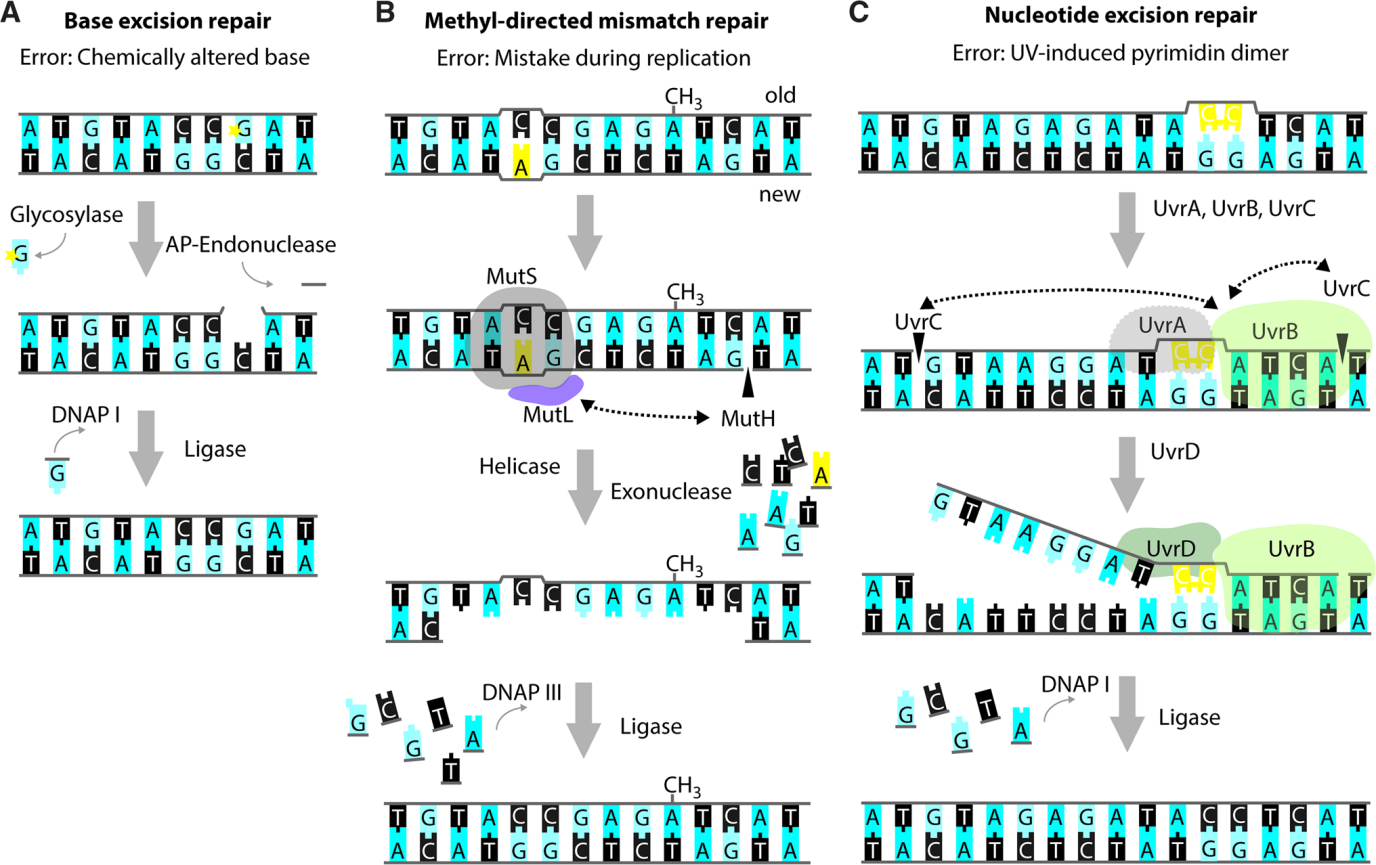
Repair of single-strand DNA lesions. Schematic representations of the *modus operandi* of base excision repair (BER) (**a**), methyl-directed mismatch repair (MMR) (**b**) and nucleotide excision repair (NER) (**c**). The lesions in the figure serve merely as examples as the aforementioned repair pathways are capable of repairing a variety of different lesions. In all examples, bases are shown as blocks using the one-letter code, the deoxyribose-phosphate moiety is depicted as a *grey line*. Incisions are indicated by *black triangles* penetrating the sugar–phosphate backbone. **a** Example of BER acting on a chemically altered base (denoted by the *yellow star*). The affected nucleotide is removed by the subsequent action of a glycosylase and an AP-endonuclease. DNAP I re-synthesizes the missing part of the DNA strand and DNA ligase closes the nick. **b** Example of MMR acting on a wrongly incorporated adenine (in *yellow*). MutS binds to the site of the distortion and subsequently recruits MutL and MutH. MutH incises the newly synthesized, non-methylated strand at the sequence GATC. Subsequently, a DNA helicase and exonuclease unwind and degrade part of the newly synthesized strand, including the non-matching nucleotide(s). DNAP III and DNA ligase fill in the missing sequence. **c** Example of NER acting on a pyrimidine dimer (in *yellow*). The UvrAB-complex binds to the site of the lesion and promotes incisions 3′ and 5′ from the lesion by UvrC. Subsequently, the UvrD-helicase promotes dissociation of the contained stretch of DNA. Also in NER, DNAP I re-synthesizes the missing part of the DNA strand, and DNA ligase closes the nick

Small chemical alterations of bases, like oxidation, deamination, alkylation, or abasic sites resulting from hydrolysis of the N-glycosidic bond between the base and the sugar moiety, are recognized and repaired by the BER system [41] (Fig. 3a). These modifications are part of the natural decay of DNA, but their occurrence is increased by e.g., the addition of a variety of chemicals and UV radiation (e.g., [42]). If unrepaired, these modifications can impact replication fidelity; e.g., oxidation of guanine yields 7,8-dihydro-8-oxoguanine (8-oxoG) that most commonly mis-pairs with adenine, resulting in a G:C to T:A transversion [41, 43, 44]. *E. coli* possesses several DNA glycosylases that recognize altered bases and mediate their removal from the DNA, thereby creating an abasic site [44, 45]. Enzymes with apurinic/apyrimidinic (AP)-endonuclease activity mediate the release of the remaining deoxyribose-phosphate moiety and the remaining gap is filled and subsequently sealed by DNAP I and DNA ligase [41, 42, 46].

The MMR system can recognize mis-paired bases directly upon replication, and short loops of non-matching nucleotides [47] (Fig. 3b). The methylation state of the DNA allows the MMR system to distinguish the newly synthesized DNA strand from the template DNA, since adenines in the symmetric sequence GATC/CTAG are methylated by the Dam methylase but remain temporarily non-methylated in the newly synthesized DNA strand [48]. Briefly, non-matching nucleotides or small indels cause a distortion, which is recognized and bound by MutS. Subsequently, MutL and MutH are recruited and the endonuclease MutH incises the most nearby, hemimethylated GATC sequence on the newly synthesized strand [49, 50]. Exonucleases then degrade the strand in both the 5′ and 3′ directions, and DNAP III fills the resulting gap [51–54]. Consistent with the role in DNA repair, strains deficient in components of the MMR system show enhanced mutation rates [55–57].

MutS and MutL are not only involved in general mismatch repair; they are also involved in VSR-mediated repair [58, 59]. The VSR system acts specifically on T:G mismatches that result from spontaneous deamination of 5-methylcytosine to thymine. Indeed, 5-methylcytosine has been shown to be a hotspot for C:G to T:A transition mutations [60]. To prevent propagation of the error and to restore the original cytosine, thymine removal is catalysed by the Vsr endonuclease [61]. The remaining gap is filled by DNAP I and DNA ligase (e.g., [58, 59, 62]).

The NER system has been shown to be active on a wide range of DNA lesions that distort DNA, e.g., UV-induced pyrimidine dimers, chemically modified bases, and, possibly, cross-links [63–65] (Fig. 3c). Upon recognition of the lesion by UvrAB, UvrC performs incisions 3′ and 5′ from the lesion. Subsequently, UvrD promotes dissociation of the contained nucleotides, and DNAP I re-synthesizes the excised sequence.

The TCR system removes lesions that hinder progression of the *E. coli* RNAP complex [65–67]. Briefly, upon RNAP stalling, recruitment of the transcription repair coupling factor, Mfd, leads to clearance of the RNAP complex from the lesion, primarily by fostering continued elongation [68]. Subsequently, components of the NER system are recruited to the lesion and repair it. Whilst the NER system acts on lesions on both DNA strands, TCR is thought to foster template strand repair upon transcription [68].

Apart from the above-mentioned repair pathways, *E. coli* has several enzymes at its disposal that directly reverse chemical alterations, like the photolyase PhrP that resolves pyrimidine dimers, the triphosphatase MutT that catalyses the conversion of 8-oxoGTP to 8-oxoGMP, or methyltransferases that take over methyl groups from alkylated bases [69–71].

### Recombination-dependent repair

In addition to the above-described lesions, *E. coli* can repair double-strand DNA breaks and single-strand DNA gaps [72]. DsDNA breaks can result from e.g., stalled replication forks at unrepaired ssDNA lesions, and single-strand gaps can result from e.g., exposure to ionizing radiation and UV light.

**Fig. 4:**
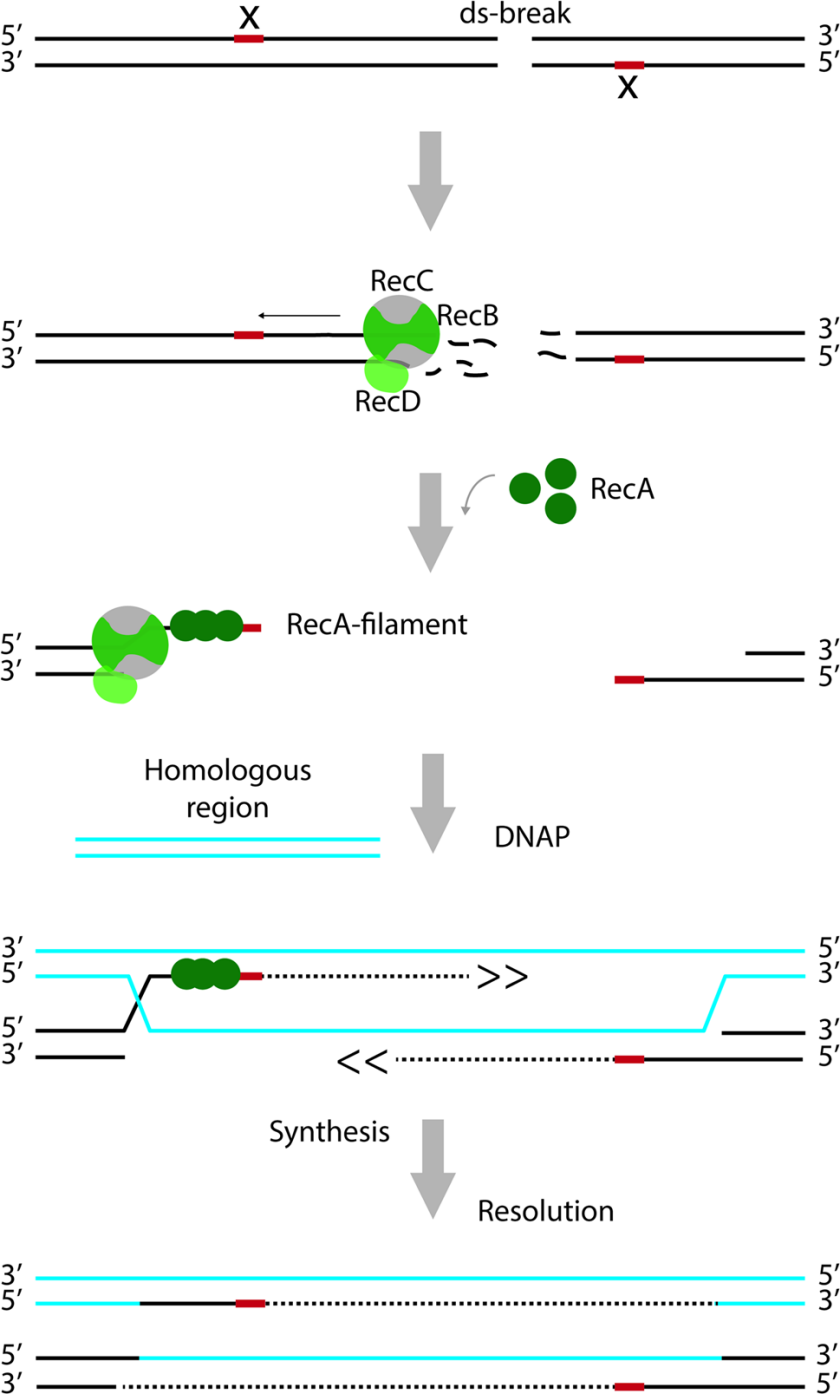
Recombination-dependent repair of double-strand breaks. The RecBCD complex has both helicase and nuclease activity. It unwinds the DNA starting from the site of the break and degrades both strands during this process. Movement of RecBCD along the DNA is indicated with an* arrow*. At specific sites (indicated by ‘*x*’), the activity of the complex is altered such that only the strand with the free 5′ end continues to be degraded. That way, a 3′ overhang is created. RecA forms a nucleoprotofilament at the 3′ overhang and promotes strand invasion at a homologous double strand. Templated by the homologous DNA, replication re-starts and the missing sequences are filled in, followed by resolution of the resulting Holliday junctions. For the sake of clarity, proteins are only depicted on one site of the double-strand break

Repair of dsDNA breaks relies on the presence of a homologous DNA sequence and the recombinase RecA (Fig. 4). First, starting from the point of the ds break, the RecBCD complex mediates unwinding of the DNA and degradation of the ssDNA strands. Specific motifs in the DNA, termed CHI-sites, alter the nucleolytic activity of the complex such that a 3′ ssDNA overhang is created [73, 74]. RecA forms a nucleoprotofilament at the 3′ overhang and mediates homology searching and strand invasion at a homologous double strand. Templated by the homologous DNA, replication re-starts and the missing sequences are filled in, followed by resolution of the resulting Holliday junctions. Just as the repair of dsDNA breaks, also post-replication repair of ssDNA gaps requires RecA-mediated strand invasion, templated DNA-synthesis and resolution. However, the initial steps are catalysed by RecFOR rather than RecBCD [75]. For detailed information on the players and steps involved in the repair of dsDNA breaks and ssDNA gaps, see e.g., [76–78]. Finally, RecA, together with components of the NER pathway, has been implicated in the repair of DNA lesions in ssDNA regions [79].

### Global response regulators and other factors affecting mutability

Throughout the previous sections, we focused on individual components and systems involved in DNA replication and repair. Importantly, all these components and systems are part of global networks that can affect mutagenesis in *E. coli* at a given condition (e.g., [77]). The number of mutations that accumulates in a strain within a defined period of time is commonly referred to as its mutation rate and depends on the selection conditions used. Mutation rates observed under conditions with minimal selection are referred to as baseline or spontaneous mutation rates. Recently, the Foster laboratory determined the spontaneous mutation rate for *E. coli* at 0.87 × 10^−3^ nucleotides per genome per generation [56]. This number includes base substitution mutations and small indels of four or less nucleotides, which constituted the vast majority of the changes observed. However, adverse conditions, like nutrient deprivation, the presence of antibiotics, exposure to certain chemicals or temperature fluctuations, can elicit (global) responses that can lead to an increase in the accumulation of mutations [37].

One of the best characterized global responses in *E. coli* that can affect the mutation rate is the SOS response (e.g., [77, 80, 81]). This response is invoked by lesions in the DNA that hinder replication and result in ssDNA stretches, and coordinates expression of many of the above-mentioned genes (e.g., [82, 83]). Briefly, under standard conditions, the repressor LexA prevents transcription of these genes by binding to a specific sequence (the SOS box) in their operator region. ssDNA stretches are bound by the recombinase RecA, which then stimulates self-cleavage of LexA. Upon self-cleavage, LexA dissociates from the SOS box, allowing transcription of the SOS genes and, subsequently, DNA repair. Importantly, the SOS response appears to be precisely timed, and coordinated and fine-tuned by a multitude of mechanisms including the aforementioned transcriptional regulation and diverse post-translational mechanisms and interactions, presumably to avoid excessive mutations (e.g., [77, 84–87]).

The RpoS response has also emerged as a key modulator of the evolution of *E. coli*. The alternative sigma factor RpoS governs the general stress response and has been proposed to affect the expression levels of more than 200 genes, including *mutS* and *mutH* (e.g., [88–90]). RpoS deficiency results in decreased levels of DNAP IV in stationary phase *E. coli* cells, indicating a role of DNAP IV in the starvation response [91]. Interestingly, it has been shown that e.g., β-lactam antibiotics can lead to an increase of mutation rates and alter the mutation pattern in an RpoS-dependent manner, likely owing to increased levels of the DNAP IV with a concomitant decrease in MutS levels [92]. Recently, the Ferenci laboratory showed that varying RpoS levels gives rise to different mutation rates and patterns. Increasing the levels of RpoS leads to a decrease of MutS levels and an increase of DNAP IV levels and vice versa [90, 92, 93]. These observations are highly relevant for the isolation of strains with improved protein production characteristics, since, during their isolation, cells are exposed to stress caused by the production of proteins.

Apart from the above-mentioned global regulators, molecular chaperones can also affect mutagenesis during the evolution of *E. coli* strains [94–96]. Indeed, several lines of evidence indicate that molecular chaperones can actually buffer mutations that compromise protein structure and function [96, 97]. It has been shown that e.g., levels of the molecular chaperones GroEL and DnaK are increased in *E. coli* strains with elevated mutation rates. In keeping with previous observations [97], expression of *groEL* mitigated the growth defects in mutated strains but had no effect on their ancestor [96]. Recently, it has been shown that RNA chaperones can also act as mutation buffers for mutations affecting RNA structure [94].

## *E. coli* as a platform for the production of recombinant proteins

*Escherichia coli* is the most widely used host to produce recombinant proteins. However, *E. coli*-based protein production can be hampered at many different levels.

It has been shown that the efficient production of proteins can be hampered by e.g., inefficient binding of the mRNA encoding the target protein to the ribosome, instability of the mRNA, secondary structures in the mRNA and non-optimal codon usage. These problems can usually be solved by modifying the target gene and its flanking regions in the expression vector [2, 98]. Protein production can also be hampered by metabolic stress (e.g., [99–102]). This will negatively affect biomass formation and, consequently, may result in insufficient production yields. It has been shown that biomass formation can be improved by e.g., changing culture and/or target gene expression conditions as well as metabolic engineering (e.g., [2, 103]). The production of sufficient amounts of functional protein can also be hampered by e.g., misfolding, degradation and mistargeting of the target protein [104]. There are examples where deleting genes encoding proteases, or overexpressing genes encoding molecular chaperones or targeting factors have led to improved protein production yields (e.g., [105–108]). Ideally, such engineering approaches to improve the production of a protein are based on detailed knowledge of what hampers its production. Unfortunately, it is usually not known what hampers the production of a protein. Therefore, engineering approaches are also used in combination with trial-and-error-based protein production screening exercises. However, to identify a strain with improved protein production characteristics, this way is often not successful.

When sufficient knowledge to engineer a strain with improved protein production characteristics is lacking, one could try to isolate such a strain using evolutionary approaches, i.e., by selecting or screening for a genetically altered strain with improved production characteristics. So far, evolutionary approaches have only been used on a very limited scale to isolate *E. coli* mutants with improved protein production characteristics. However, they have been widely and successfully used to modulate metabolic pathways in *E. coli* for metabolite production (e.g., [109–111]). This indicates that the use of evolutionary approaches to isolate *E. coli* protein production strains may actually have more potential than currently appreciated.

**Fig. 5:**
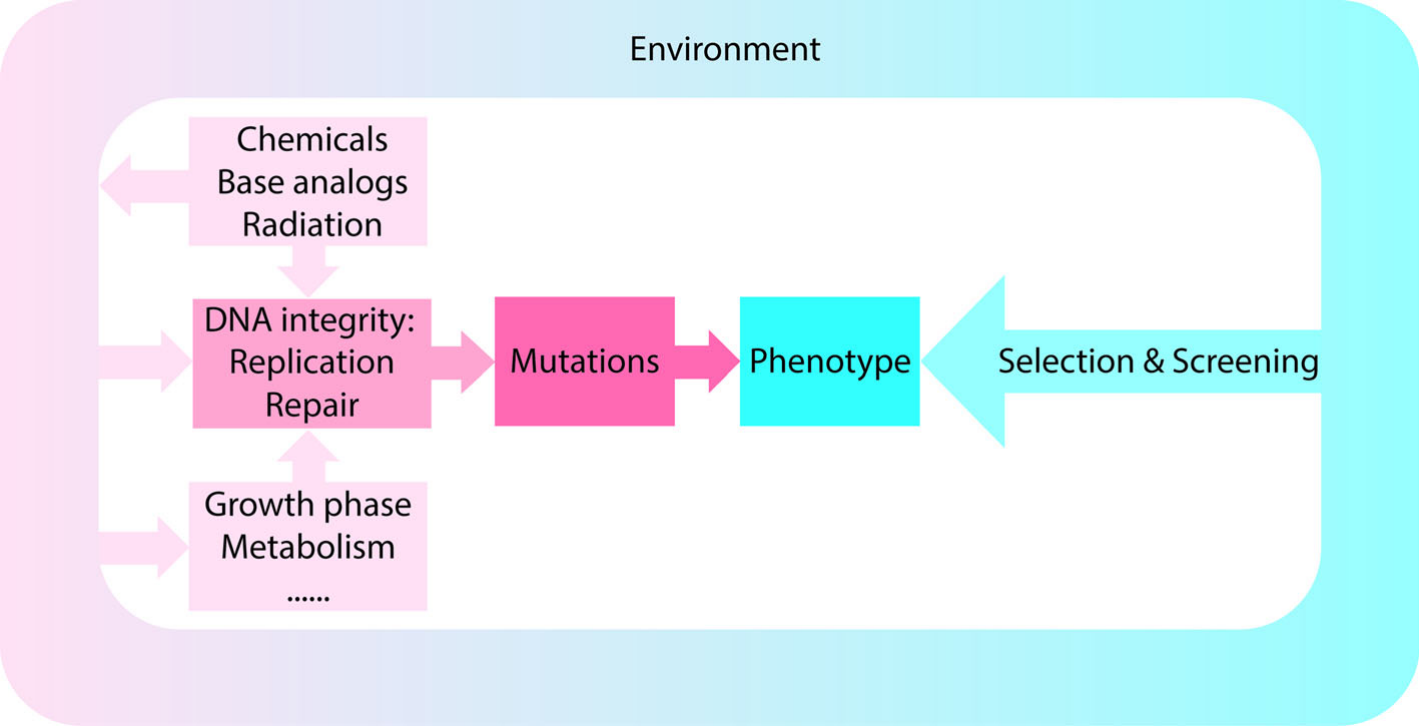
Factors affecting mutation rates and patterns. Schematic representation of how extrinsic and intrinsic factors may contribute to the observed mutation rates and patterns. Screening for or selection of a certain phenotype is based on the acquired mutations

In a selection, conditions are used in which only the desired mutant can multiply or its multiplication is at least strongly favored [4]. In contrast, in a screen, a large number of bacteria are examined under non-selective conditions to identify the strain with the desired characteristics [4, 112]. Both approaches rely on mutations, and as described above and summarized in Fig. 5, mutation rates and spectra and, thus, the outcome of the isolation, can be affected by a variety of different factors.

In the following sections, we will discuss the, in our view, most relevant examples of the isolation of evolved *E. coli* strains with improved protein production characteristics. We will first focus on approaches that employed mutagenic agents, mutator genes or transposons to facilitate mutagenesis. Subsequently, we will discuss isolation strategies relying on spontaneous mutations. Finally, we will discuss how evolutionary approaches may be used in the future to isolate *E. coli* protein production strains.

### Evolving protein production strains using mutagenic agents and mutator genes

To evolve strains with improved membrane protein production characteristics, the Bowie laboratory used an elegant selection-based approach [27]. The aim was to produce target membrane proteins in the cytoplasmic membrane rather than in inclusion bodies, since it is relatively easy to isolate membrane proteins from a membrane system compared to isolating them from inclusion bodies [113]. It should be noted that when a membrane protein is inserted into a membrane system, it does not necessarily mean it is properly folded and functional. The gene encoding the membrane protein of interest was cloned in two compatible expression plasmids. Each plasmid was constructed such that the membrane protein was C-terminally fused to an antibiotic resistance marker conferring resistance to trimethoprim (plasmid 1) or kanamycin (plasmid 2). This way, an increased resistance to the two antibiotics could be used as a direct indicator for elevated levels of the target protein inserted in the cytoplasmic membrane.

To introduce (chromosomal) mutations, cells containing plasmid 1 were either exposed to the mutagenic base analog 2-aminopurine (AP2), which is an adenine analog that can miss-pair occasionally with cytosine, or they were transformed with an expression plasmid containing the mutator gene *mutD5*, which encodes a variant of the ε subunit of DNAP III that is deficient in 3′–5′ exonuclease activity [114]. Subsequently, mutant strains with increased resistance towards trimethoprim were selected for. Positive candidates were then transformed with plasmid 2 and probed for increased resistance towards kanamycin. Importantly, the use of a dual selection strategy considerably lowered the risk of obtaining unrelated mutations that confer resistance to both antibiotics without increasing membrane protein production yields. Several mutant strains that produced elevated levels of the target membrane proteins were isolated. Interestingly, these strains differed widely in their ability to produce membrane proteins other than the target used during their isolation. Unfortunately, these strains have never been characterized in detail, and the mutations underlying their phenotypes are still unknown. Given that the mutation rates were increased using a base analog or a mutator gene, it is very well possible that such an analysis would be complicated due to the presence of non-related mutations. Indeed, the authors reported a 300-fold increase in mutation rates over background upon using the *mutD5* allele. It should be noted that one of the isolated strains showed a reduced copy number of the expression plasmids used. It has been speculated that the reduced plasmid copy number may lead to lowered target gene expression intensity, thereby improving membrane protein production yields in the cytoplasmic membrane [115].

The Georgiou laboratory employed chemical mutagenesis to generate *E. coli* mutants that efficiently produce properly assembled full-length IgG antibodies in the periplasm [116]. The periplasm is the preferred compartment of the *E. coli* cell to produce disulfide bond-containing proteins like IgG antibodies. In contrast to the reducing cytoplasm, disulfide bonds can be stably formed in the periplasm due to the presence of DsbA and DsbB [117]. The periplasmic protein DsbA harbours a thioredoxin domain and acts as oxidizing agent (electron-acceptor) for the disulfide bond-forming cysteines of the target protein. The cytoplasmic membrane protein DsbB receives electrons from DsbA and transfers them to quinones in the cytoplasmic membrane, thereby maintaining DsbA in an active state. Cells harbouring an IgG expression plasmid were treated with the alkylating agent *N*-methyl-*N’*-nitro-*N*-nitrosoguanidine (MNNG), and clones with increased levels of functional IgG were isolated using a small, fluorescent IgG antigen and repeated rounds of fluorescence-activated cell sorting (FACS). Note that MNNG mainly methylates guanines at the O^6^ position. The resulting *O* ^6^-methylguanine base pairs with thymine during DNA replication, which may result in a G:C to A:T transition [118]. Using this approach, the authors isolated several clones that were markedly improved in their ability to produce IgGs. Also here, the defining mutations were not identified. In this respect, it should be noted that MNNG can increase the mutation frequency several hundred fold above background level [119]. Therefore, it is possible that the isolated strains have acquired both beneficial and deleterious mutations in multiple loci, which might hinder identifying the mutations responsible for the improved production of IgG. Moreover, the ancestral strain was deficient in the recombinase RecA, one of the major players in recombination-dependent DNA repair. Indeed, most of the isolated clones were excluded from further studies due to growth defects, indicating the accumulation of deleterious mutations.

Recently, Hatahet et al. isolated *E. coli* mutant strains that efficiently produce a variant of the mammalian polytopic membrane protein vitamin K epoxide reductase (VKORc1) [120]. Mammalian VKORc1 maintains thioredoxin-like proteins in an oxidized state by transferring electrons to membrane-bound quinones, analogous to the previously described DsbB. Despite these functional analogies, mammalian VKORc1 could not restore motility of an *E. coli* strain lacking *dsbB*. In *E. coli*, DsbB is critical to mediate an essential structural disulfide bond in FlgI, a major component of the flagella machinery [121]. Therefore, first, a VKORc1 variant that is functionally produced in *E. coli* was isolated. To this end, a mutagenized *vkorc1* expression plasmid library was created using the mutator strain XL1-Red [122]. This strain contains the aforementioned *mutD5* allele and is deficient in both MutS, which is involved in several DNA repair pathways, and MutT, which counteracts replication errors that may arise from the oxidation of guanine (see above). Using the mutagenized expression library, functional VKORc1 variants (mtVKORc1) were selected for based on their ability to (partially) restore motility of a strain lacking *dsbB*.

Since the isolated mtVKORc1s only partially restored the ability to form disulfide bonds in the DsbB-deficient strain, strains with improved mtVKORc1 production characteristics were isolated. *E. coli* cells were treated with the mutagenic agent ethyl methanesulfonate (EMS) to facilitate mutagenesis [114]. Similar to the aforementioned mutagenic agent MNNG, EMS preferentially alkylates guanine at the O^6^ position which can result in G:C to A:T transitions. From the treated cells, mutants with improved production levels for one of the mtVKORc1s were selected on plates containing the disulfide-breaking agent, Tris 2-carboxyethyl phosphine, that prevents growth of strains deficient in disulfide bond formation. In the isolated clones, disulfide bond formation, i.e., production levels of functional mtVKORc1, was subsequently probed using different phenotypic screens.

Sequencing of 11 strains that produced increased levels of functional mtVKORc1 revealed that they had accumulated multiple mutations in different locations. However, seven out of the eleven isolated strains had acquired a non-synonymous base substitution in the gene encoding the membrane insertase/foldase YidC, indicating a potential benefit. Indeed, three out of the four different mutations led to a roughly fourfold increase of the levels of functional mtVKORc1. Interestingly, two of the mutations that increased mtVKORc1 yields resulted in an amino acid exchange in the hydrophilic groove of YidC, which is the part of the membrane-integral portion of the protein that has been implicated in substrate binding [123]. This led the authors to suggest that, at least in the case of these mutations, higher functional yields of mtVKORc1 may be attributed to a more relaxed YidC substrate binding specificity. Besides the mutations in *yidC*, also mutations inactivating HslV, the protease subunit of the cytoplasmic HslUV complex, were identified and shown to enhance production yields of both mtVKORc1 and VKORc1, possibly by preventing their premature degradation in the cytoplasm. However, activity increased only for mtVKORc1, highlighting the need to monitor protein activity when improving protein production yields.

Combining proteolytically inactive HslV with mutated YidC further improved the functional yields of mtVKORc1. Using a *yidC* mutant strain also decreased toxicity of proteorhodopsin production was observed [120]. However, deletion of *hslV* had the opposite effect, illustrating that the outcome of a genetic alteration can be highly context-dependent.

### Transposon mutagenesis

Also, transposon mutagenesis has been used to isolate strains with improved protein production characteristics. It should be kept in mind that the nature of mutations caused by transposons is different than the ones caused by mutagenic agents and mutator genes, although the effect can be similar [40]. Insertion of a transposon in the vicinity of a gene can affect its expression levels, and insertion into an intact gene may result in complete loss of function or lead to the synthesis of a truncated variant of the encoded protein, thereby affecting its function. However, in contrast to approaches based on mutagenic agents and mutator genes, identifying the *loci* targeted by a transposon is relatively straightforward, which facilitates identifying the genetic basis of the improvement [20, 21, 112].

The Georgiou laboratory used Tn*5* transposon mutagenesis to isolate *E. coli* variants that produce increased amounts of the human GPCR central cannabinoid receptor (CB1) in the cytoplasmic membrane [124]. Due to a lack of any noticeable insertion sequence preference, Tn*5* is a widely used mobile element for approaches relying on transposon mutagenesis [125]. To monitor CB1 production levels in the cytoplasmic membrane, the protein was fused to green fluorescent protein (GFP) [126]. This enabled enriching the cells with improved CB1–GFP production characteristics from a pool of Tn*5* insertion mutants using FACS. Subsequently, single clones were isolated and the site of Tn*5* insertion was determined. The most pronounced improvement in CB1–GFP accumulation levels, as well as in biomass formation, was found to be due to a Tn*5* insertion in the gene encoding DnaJ, which is a co-chaperone that is part of the DnaK/DnaJ/GrpE chaperone system [127]. Interestingly, Tn*5*::*dnaJ* did not improve the production of any of the other GPCRs tested. Thus, Tn*5*::*dnaJ* specifically improved CB1 production, and furthermore, the improvement did not depend on the presence of the GFP moiety. To explain the observed phenotype, the authors hypothesized that the absence of DnaJ may either increase the efficiency at which CB1 is targeted to the membrane or, alternatively, prevent the DnaK/DnaJ-mediated degradation of CB1. However, it is also possible that the effects of Tn*5*::*dnaJ* are considerably more pleiotropic and even influenced by the temperature shift from 37 to 12 °C during the production of CB1. In mutants carrying a defective *dnaJ* allele, the heat-shock response is continuously ON, due to impaired regulation of the heat-shock sigma factor σ^32^ [128]. DnaJ has been shown to interfere with *lon*-mediated degradation of secretory proteins, and the absence of *dnaJ* has been suggested to delay ribosome biosynthesis [129, 130]. All this makes that it may be very difficult to elucidate how *dnaJ* mutations improve the production of CB1.

*Escherichia coli* naturally secretes the YebF protein into the extracellular medium and it has successfully been used as a fusion partner for the production of recombinant proteins in the extracellular medium [131, 132]. Haitjema et al. used Tn*5* transposon mutagenesis to isolate mutants with improved secretion characteristics for YebF/YebF fusion proteins [133]. To rapidly screen for such mutants, a fluorescence-based assay enabling to specifically detect YebF secreted into the extracellular medium was used. Eight different gene insertions leading to improved secretion of YebF/YebF fusion proteins into the extracellular medium were isolated. For two mutants, one with the *envZ* gene and the other with the *ompR* gene disrupted by Tn*5*, it was shown that the cell envelope was less stable, presumably leading to the leakage of proteins into the extracellular medium. For the remaining six mutations, the mechanism leading to enhanced secretion of YebF/YebF fusion proteins into the extracellular medium remains speculative.

Finally, Tn*5* transposon mutagenesis was also used to isolate *E. coli* variants that produce increased amounts of signal recognition particle (SRP)-targeting pathway-dependent secretory proteins and membrane proteins in the periplasm and cytoplasmic membrane, respectively [134]. In *E. coli*, the SRP-targeting pathway guides a subset of secretory proteins and most membrane proteins, in a co-translational fashion, to the protein conducting Sec-translocon in the cytoplasmic membrane [135]. To allow rapid screening of a Tn*5* transposon-insertion library for clones with improved protein production characteristics, the authors used a fluorescence-based assay to monitor protein production in the periplasm. In all analysed mutants, Tn*5* had disrupted the *rrsE* gene, which is one of the seven gene copies in *E. coli* encoding the 16S rRNA. The *rrsE* deficiency was shown to improve the periplasmic production of proteins secreted via the SRP-targeting pathway and the production of membrane proteins in the cytoplasmic membrane. The underlying mechanism for this improvement is yet unknown. Notably, it was shown that the *rrsE* deficiency not only leads to increased production yields in standard batch cultures, but also in high cell density fermentations.

### Isolation of protein production strains without facilitating mutagenesis

On a limited scale, strains with improved protein production characteristics have also been isolated without facilitating mutagenesis. Nevertheless, the procedures used to isolate these strains may very well have affected mutation rates and patterns (Fig. 5). Probably, the best known examples of protein production strains isolated without facilitating mutagenesis are the BL21(DE3)-derived C41(DE3) and C43(DE3) strains [136]. We will first discuss their isolation and then give an overview of the other reported examples.

### The isolation of C41(DE3) and C43(DE3)

C41(DE3) was isolated from the common protein production strain BL21(DE3), and C43(DE3) was subsequently isolated from C41(DE3) [136]. In BL21(DE3), expression of the gene encoding the target protein is driven by bacteriophage T7 RNA polymerase (P), which transcribes eight times faster than *E. coli* RNAP [137–139]. T7 RNAP specifically recognizes the T7 promoter, which drives the expression of the target gene from a plasmid [137, 139]. The gene encoding the T7 RNAP is under control of the *lac*UV5 promoter region (P_*lac*UV5_), which is a strong, carbon-catabolite repression protein (CRP)–cAMP independent variant of the wild-type *lac* promoter region (P_*lac*WT_) [140, 141]. Note that we chose the term ‘region’ to incorporate sequence differences observed in the CRP–cAMP binding site and the O1 operator site in BL21(DE3). The addition of isopropyl-β-d-thiogalactopyranoside (IPTG) leads to the production of T7 RNAP and, consequently, expression of the target gene. Expression of genes encoding recombinant proteins is often toxic to BL21(DE3), resulting in poor growth and low protein production yields. Major reasons for this toxicity appear to be the saturation of protein biogenesis pathways and metabolic stress (e.g., [100, 101]).

**Fig. 6:**
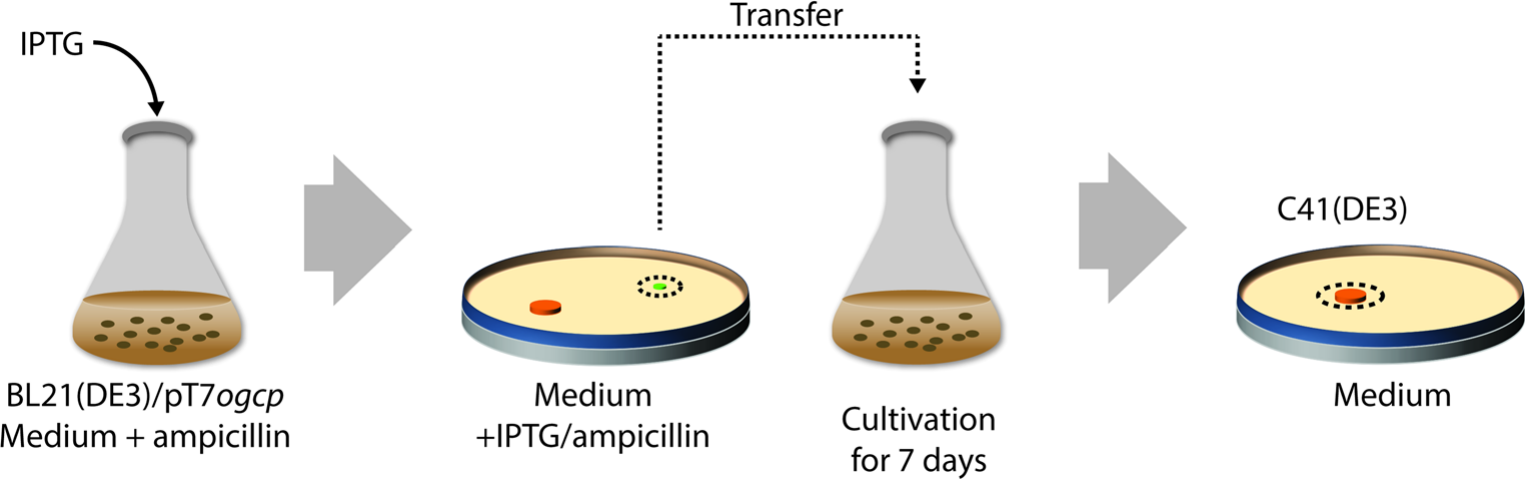
Isolation of C41(DE3) from BL21(DE3). To isolate C41(DE3), BL21(DE3) was first transformed with a T7-based expression vector harbouring the gene encoding the mitochondrial oxoglutarate malate carrier protein (OGCP) and expression of *ogcp* was induced with IPTG in liquid culture. Notably, the *ogcp* expression vector has an ampicillin resistance marker. Surviving cells were selected for on IPTG-containing agar plates and subsequently probed for efficient OGCP production. In a second step, selected clones were cured from the *ogcp* expression vector by culturing them for a prolonged period of time in a closed setup (modified after [143])

**Fig. 7:**
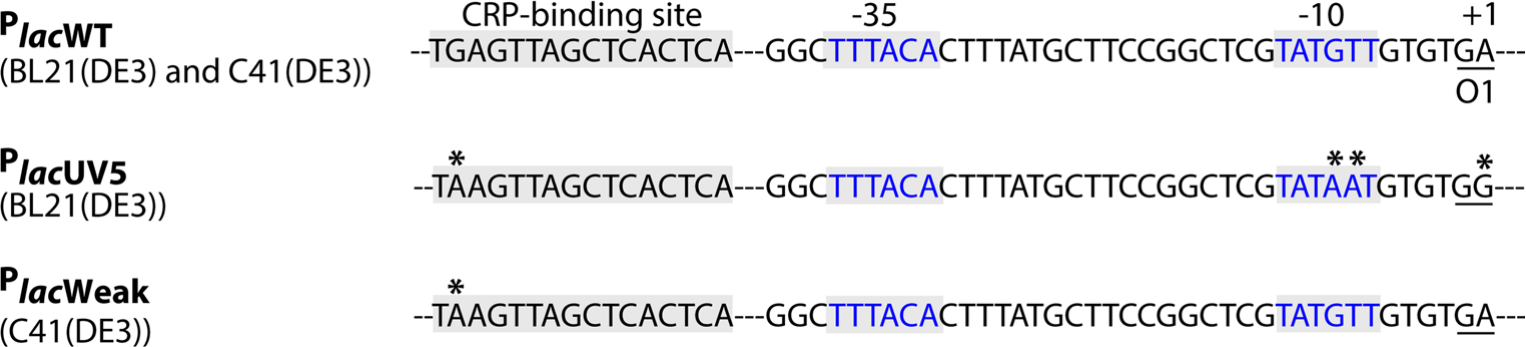
P_*lac*WT_, P_*lac*UV5_ and P_*lac*Weak_. Expression of the *lac* operon (*lacZYA*) is governed by the P_*lac*WT_ region. A variant of this well-known promoter region, termed P_*lac*UV5_, controls the expression of the gene encoding T7 RNAP in BL21(DE3) [142]. This variant differs from P_*lac*WT_ in four positions (*asterisk*). For better orientation, we highlighted the relevant sites: the binding site for CRP–cAMP, the −35/−10 binding sites for *E. coli* RNAP, and the first bases of the O1-operator site. Note that the term region was chosen to account for all four mutations. In different BL21(DE3)-derived protein production strains including C41(DE3) [101, 142, 143, 146], P_*lac*UV5_ has reverted to a weaker variant, designated P_*lac*Weak_ [143]. This variant still harbours the altered CRP–cAMP binding site of the P_*lac*UV5_ region, but reverted to P_*lac*WT_ in the −10 and the O1-operator site Picture was taken from [143] with permission

To isolate C41(DE3), BL21(DE3) was transformed with a T7-based expression vector harbouring the gene encoding the mitochondrial oxoglutarate malate carrier protein (OGCP) [136] (Fig. 6). Expression of *ogcp*, which is highly toxic, was induced with IPTG, and surviving cells were selected for, on IPTG-containing agar plates. Thus, toxicity of *ogcp* expression served as selective agent. In a second step, IPTG-resistant clones that efficiently produced OGCP were cured from the *ogcp* expression plasmid by culturing them for a prolonged period of time in a closed setup. This led to the isolation of C41(DE3), which not only can efficiently produce OGCP but also many other proteins whose production is toxic to BL21(DE3). Recently, it was shown that three single nucleotide polymorphisms (SNPs) in P_*lac*UV5_ are solely responsible for the improved protein production characteristics of C41(DE3) [101, 142, 143]. The three SNPs specifically change the −10 region and the O1-operator/+1 site of P_*lac*UV5_ to P_*lac*WT_ (Fig. 7). This weakens the promoter region, resulting in reduced *t7rnap*- and, consequently, target gene expression levels upon addition of IPTG. Notably, the promoter region governing *t7rnap* expression in C41(DE3) appears not only to be weaker than P_*lac*UV5_ but also weaker than P_*lac*WT_ and was, therefore denoted P_*lac*Weak_ [143]. Although, in first instance, counterintuitive, reduced target gene expression levels result for many target proteins in higher protein production yields, because the overloading of the protein biogenesis machinery as well as metabolic stress are reduced [101, 144].

A pivotal experiment in the identification of the defining mutations of C41(DE3) was the reconstruction of its isolation from BL21(DE3) in real time [143]. This approach revealed that BL21(DE3) derivatives harbouring the same three SNPs in P_*lac*UV5_ as C41(DE3) could be isolated within only a couple of hours after the transformation of the *ogcp* expression vector into BL21(DE3). Both the speed of the occurrence of the mutations in P_*lac*UV5_ and their specific nature could be best explained by homologous recombination between P_*lac*UV5_ and P_*lac*WT_ that is part of the *lac* operon present in BL21(DE3). Recombination is most likely facilitated by the presence of sizable DNA sequences around P_*lac*UV5_ governing *t7rnap* expression that are homologous to the ones flanking P_*lac*WT_ in the *lac* operon. Indeed, mutations in P_*lac*UV5_ did not occur in BL21(DE3) derivatives that are *recA*-deficient or lack P_*lac*WT_ in the *lac* operon. Using expression vectors containing genes encoding target proteins other than OGCP gave similar results, and for some targets, even BL21(DE3) derivatives with a complete conversion of P_*lac*UV5_ to P_*lac*WT_ were isolated [143]. The accumulation of mutations in P_*lac*UV5_ represents an interesting case of evolutionary trade-off. On the one hand, they provide an easy and very fast, if not the fastest possible, escape from the immediate toxicity of protein production and, therefore, a large, initial growth benefit. On the other hand, these cells still produce the target protein, which should reduce their growth relative to non-producers, and consequently, they may be outcompeted by the non-producers over time.

Even though many difficult proteins could be efficiently produced in C41(DE3), there were some exceptions, like the subunit b of the *E. coli* F_o_F_1_ ATPase (Ecb) [136]. This membrane protein was used to isolate C43(DE3) from C41(DE3) following essentially the same experimental setup used for the isolation of C41(DE3) from BL21(DE3) [136]. Mutations in the *lac*-repressor gene, *lacI*, adjacent to the gene encoding the T7 RNAP appear to be key to the improved protein production characteristics of C43(DE3) [142]. It has been proposed that these mutations result in a LacI variant that binds with a higher affinity to the *lac* operator site. This is in keeping with lowered T7 RNAP accumulation levels in C43(DE3) compared to C41(DE3), and with the delayed onset of the synthesis of the lactose permease, LacY, in C43(DE3) upon the addition of IPTG [101]. Interestingly, it seems that the mutations in *lacI* could only be selected for when P_*lac*Weak_ was governing *t7rnap* expression [142].

Both C41(DE3) and C43(DE3) acquired additional mutations during their isolation [142, 143]. The role of many of these mutations is not clear yet. Some of the mutations enhance the ability to take up nutrients in C41(DE3). It has, therefore, been suggested that they can be attributed to starvation stress imposed during the plasmid curing step [143]. It is worth mentioning that five out of the 12 changes specific for C43(DE3) and one alteration common to both C41(DE3) and C43(DE3) involve IS-elements [142, 143]. Since the activity of IS-elements has been linked to stressful conditions, at least part of the observed alterations might be a consequence of the isolation procedures used. It is also worth mentioning that in C43(DE3), expression of *lon* is restored [101, 142]. The Lon protease interferes quite often with the production of proteins in the cytoplasm and BL21(DE3) is *lon*-deficient due to an IS-element inserted in the *lon* promoter region. In C43(DE3), *lon* expression is restored due to the removal of this IS-element and this could explain why some proteins are produced less efficiently in C43(DE3) [101, 136, 142] (see below). However, as observed by Hatahet et al., protease deficiency not necessarily positively affects the production of functional protein material and it is very well possible that the production of some targets benefits from the restored Lon activity due to an increased turnover of misfolded/aggregated proteins.

### Other examples of the isolation of protein production strains without facilitating mutagenesis

To the best of our knowledge, so far, four other examples of the isolation of *E. coli* strains with improved protein production characteristics without facilitating mutagenesis have been reported.

Zhao et al. used a *recA*-deficient BL21(DE3) derivative to isolate variants that efficiently produce an engineered, bispecific single-chain antibody [145]. Similar to the setup used to isolate C41(DE3) and C43(DE3), cells containing a T7-based expression vector were spread on agar plate containing IPTG to select for IPTG-resistant mutants. Subsequently, the production of the antibody was monitored in the isolated IPTG-resistant mutants. In two of the mutants, protein production yields were about twofold higher compared to the yield obtained in their ancestor, and both mutants showed improved plasmid stability. The causative mutations have not been identified, but phenotypic differences between the two isolates suggest the presence of distinct or unrelated mutations. It would be interesting to characterize these isolates in more detail. Importantly, the use of a *recA*-deficient strain prevents a recombination-mediated event weakening P_lacUV5_. Thus, these strains most likely have acquired mutations other than the defining ones in C41(DE3) and C43(DE3).

The Cole laboratory isolated BL21(DE3)-derived mutants with an improved ability to produce the cytoplasmic *E. coli* protein CheY, which is involved in chemotaxis [146]. To facilitate monitoring protein production levels during the isolation process, CheY was fused to GFP. Expression of the *cheY*–*gfp* fusion was induced with IPTG, and GFP fluorescence was used to identify mutants with improved CheY–GFP production characteristics both on agar plates and using FACS. All mutants with improved CheY–GFP production yields had acquired the same mutations in P_*lac*UV5_ as C41(DE3). However, at least one of the mutants likely harboured additional, unidentified mutations, as CheY–GFP production yields and the fraction of soluble CheY–GFP were higher as compared to C41(DE3). The neisserial outer membrane lipoprotein Ccp, which is a cytochrome *c* peroxidase, could only be produced to very low levels in this mutant as compared to C41(DE3) and C43(DE3), strongly indicating that the effects of any unidentified mutation are target protein-dependent. Interestingly, in C43(DE3), hardly any cytoplasmic CheY–GFP was produced, whereas functional yields of the secreted, neisserial target were very high. These observations may very well be explained by the restored expression of *lon* in C43(DE3) (see above). The performance of isolated strains was also tested in more industry-scale batch cultures.

Gul et al. isolated several mutants with improved membrane protein production characteristics [147]. To this end, two target membrane proteins, the *E. coli* glutamate transporter GltP and the *Lactococcus lactis* amino acid permease BcaP, were used. The two targets were fused to both GFP, enabling monitoring the accumulation levels of the target protein in the cytoplasmic membrane, and EmrC, conferring resistance to erythromycin. Mutant strains were selected for by gradually increasing the erythromycin concentration in the culture medium. GFP fluorescence was used to monitor if increased erythromycin resistance also led to increased membrane protein production yields in the cytoplasmic membrane. Besides few other mutations, all isolated strains had acquired at least one mutation in the *hns* gene. In general, the parallel isolation of mutations in the same gene can be a first indicator for a correlation to the obtained phenotype. However, H-NS is a DNA-binding protein implicated in transcriptional repression (silencing) as well as in bacterial chromosome organization [148]. Notably, the isolated mutants varied in their ability to efficiently produce different target proteins, and data shown for at least one of the isolated strains suggest the presence of mutations specific towards the target protein used during the isolation. Given the broad range of effects that mutations in *hns* may cause and the potential contributions from other mutations, it remains unclear why the isolated strains have improved membrane protein production characteristics.

**Fig. 8:**
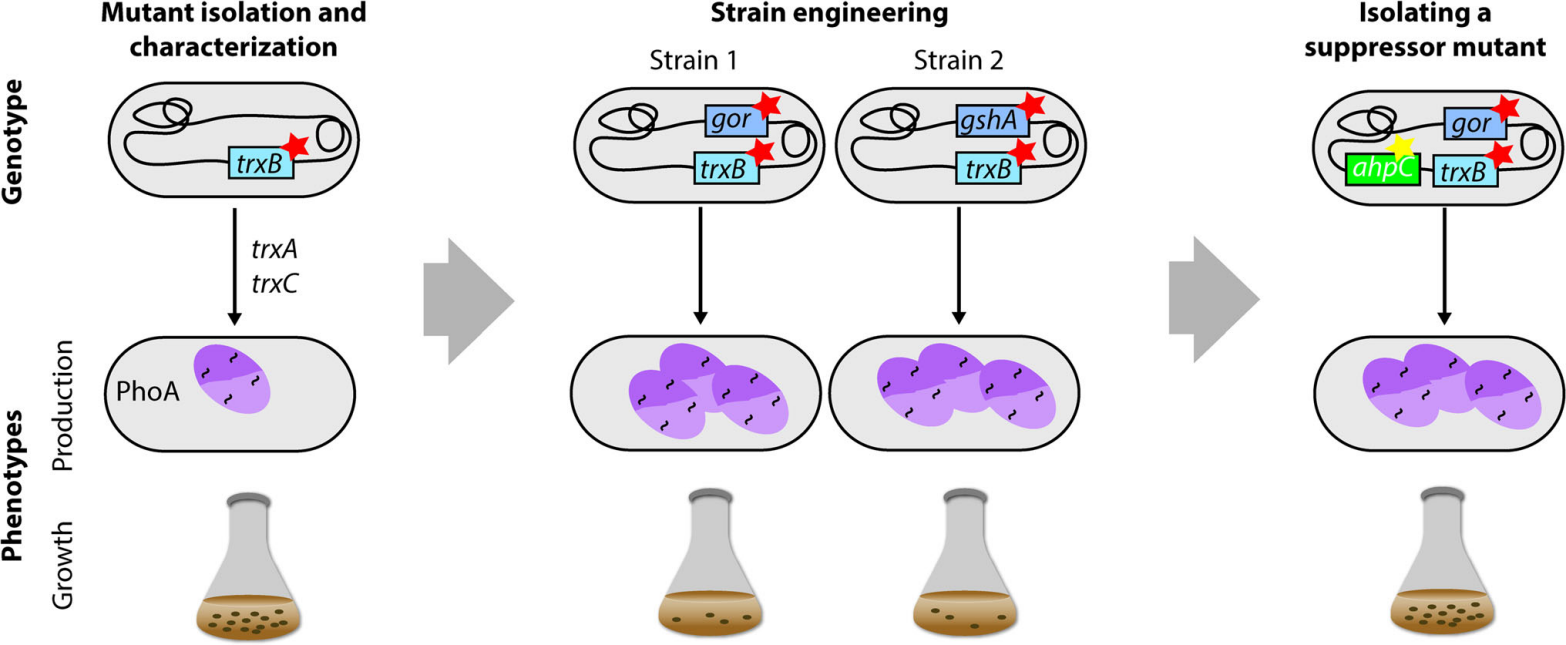
Combining evolutionary approaches and engineering to create *E. coli* strains enabling the efficient production of disulfide-containing proteins in the cytoplasm. A screening approach was used to isolate *E. coli* strains that allow the formation of disulfide bonds in the cytoplasm [150]. In the screen, PhoA, a periplasmic protein, which requires disulfide bonds for its activity, was produced without a signal sequence in a strain lacking chromosomal *phoA*. The activity of the signal-sequence-less PhoA served as an indicator for cytoplasmic disulfide bond formation. Subsequently, mutants with PhoA activity were screened for, which resulted in the isolation of *trxB*-deficient strains. Using an engineering approach, it was found that disulfide bond formation in the cytoplasm is even more efficient in *trxB* null mutants that are unable to either synthesize or reduce gluthathione (*gshA* ^−^ or *gor* ^−^) [151]. However, these double mutants grow very poorly and require an exogenous reductant to achieve a reasonable growth rate. Finally, suppressor strains were isolated that grow well and still allow stable disulfide bond formation in the cytoplasm [152]

The Beckwith laboratory used a combination of evolutionary approaches and directed engineering to create strains enabling the efficient production of disulfide-containing proteins in the cytoplasm [149] (Fig. 8). Notably, the initial aim was not to isolate protein production strains, but rather to investigate the mechanisms that prevent the stable formation of disulfide bonds in the cytoplasm. For that purpose, a screening approach was used to isolate *E. coli* strains that allow the formation of disulfide bonds in the cytoplasm [150]. In the screen, PhoA, a periplasmic protein which requires disulfide bonds for its activity, was produced without a signal sequence in a strain lacking the chromosomal copy of *phoA*. The activity of the signal-sequence-less PhoA served as an indicator for cytoplasmic disulfide bond formation. Subsequently, mutants with PhoA activity were screened for, which resulted in the isolation of *trxB*-deficient strains. TrxB encodes a thioredoxin reductase that serves as reducing agent for the two thioredoxins TrxA and TrxC. In a *trxB*-deficient mutant, the two *E. coli* thioredoxins TrxA and TrxC remain in an oxidized state and can catalyse the formation of disulfide bonds in the cytoplasm. Later on, disulfide bond formation was found to be even more efficient in *trxB* null mutants that were unable to either synthesize or reduce gluthathione (*gshA* or *gor*). However, these double mutants grow very poorly and require an exogenous reductant such as DTT to achieve a reasonable growth rate [151]. Finally, to circumvent the growth defect, suppressor strains were isolated that grow well and still allow stable disulfide bond formation in the cytoplasm [152]. These strains have been widely used to produce disulfide bond-containing recombinant proteins. Production yields can be further improved by expressing the gene encoding a disulfide bond isomerase in the cytoplasm [152].

## Concluding remarks

There is only a limited number of examples of *E. coli* strains with improved protein production characteristics isolated by evolutionary approaches. Interestingly, using evolutionary approaches to modulate metabolic pathways in *E. coli* for metabolite production has been very successful [110, 111]. This appears to be due to the relative ease to monitor most metabolites. Therefore, it is not surprising that the major bottleneck hampering the use of evolutionary approaches to isolate *E. coli* protein production strains seems to be the ability to rapidly and accurately monitor not only the amount, but also the quality of a produced protein. The importance of being able to monitor both protein quantity and quality is nicely illustrated by the isolation of strains with improved IgG and mtVKORc1 production characteristics [116, 120]. We reckon that the development of, in many instances target-specific, assays to rapidly monitor protein production will be key for extending the use of evolutionary approaches to isolate *E. coli* protein production strains.

In all the reported examples of the isolation of *E. coli* protein production strains, different strain backgrounds, promoter systems, induction regimes, culturing conditions and, sometimes, also ways to facilitate mutagenesis were used. This makes that it is currently impossible to formulate general rules for how to design an experiment to isolate an *E. coli* protein production strain. So far, it appears that the effects that most of the aforementioned factors can have on the isolation of protein production strains may have been underestimated. One obvious example is the isolation of C41(DE3) from BL21(DE3), which depended on the presence of the native *lac* wild-type promoter and RecA in the ancestor strain BL21(DE3). However, also more subtle factors like the induction kinetics of a chosen promoter system or varying levels of global regulators in different strain backgrounds may influence the evolutionary trajectories of protein production strains. Also, stress caused by e.g., starvation, temperature fluctuations, and exposure to antibiotics during the strain isolation procedure may affect evolutionary trajectories [37]. Therefore, accumulated mutations in an isolated protein production strain may not only reflect adaptations improving protein production. The presence of the same or similar mutations in multiple, parallel isolated strains may help to identify the key mutations for improving protein production or simply reflect the isolation conditions used [120, 136, 142, 143]. Incorporating temporal resolution, or evolving strains in parallel without the expression plasmid or with an empty expression plasmid may aid in discerning the accumulated mutations. It might also be interesting to see how the ability to fine-tune mutation rates and patterns could affect the isolation of protein production strains [153]. Finally, newly developed evolutionary approaches that enable to randomly alter specific loci like multiplex automated genomic engineering (MAGE) have been successfully used to isolate *E. coli* strains for metabolite production [154]. Recently, it was also used to create a strain for the efficient production of a protein containing multiple non-natural amino acids [155]. The ability to efficiently mutate defined regions in the genome may make MAGE a very powerful tool to isolate protein production strains, in particular ones where one already knows what components, e.g., molecular chaperones and protein targeting factors, or even parts thereof to target.

Surprisingly, there are only two reported examples of evolved protein production strains whose performance was also tested in a culturing setup resembling one often used in industry [134, 146]. If an *E. coli* protein production strain performs well in small batch culture, it will not necessarily also perform well in more industrial settings, like high cell density fermentations [108]. It is also possible that mutations cannot be stably maintained when changing culturing setups. Thus, if one plans to use an evolved protein production strain in a more industrial setting, one may want to test its performance in such a setting early on. However, even if changing culturing setups leads to instability, it should be kept in mind that it still may be possible to isolate suppressors alleviating the instability [108].

Taken together, we envisage that the number of examples of protein production strains isolated using evolutionary approaches will grow steadily and that, in many instances, strains will be isolated for specific target proteins. Once the use of evolutionary approaches to isolate protein production strains is more established, combining evolutionary with directed engineering approaches may very well open up avenues for the creation of the next generation of *E. coli* protein production strains.
